# Vacuum ultraviolet coherent undulator radiation from attosecond electron bunches

**DOI:** 10.1038/s41598-021-93640-8

**Published:** 2021-07-16

**Authors:** Enrico Brunetti, Bas van der Geer, Marieke de Loos, Kay A. Dewhurst, Andrzej Kornaszewski, Antoine Maitrallain, Bruno D. Muratori, Hywel L. Owen, S. Mark Wiggins, Dino A. Jaroszynski

**Affiliations:** 1 SUPA, Department of Physics, University of Strathclyde, Glasgow, UK; 2 Cockcroft Institute, Warrington, UK; 3 Pulsar Physics, Eindhoven, The Netherlands; 4 The University of Manchester, Manchester, UK; 5 STFC Daresbury Laboratory, Daresbury, UK

**Keywords:** Plasma-based accelerators, Lasers, LEDs and light sources

## Abstract

Attosecond duration relativistic electron bunches travelling through an undulator can generate brilliant coherent radiation in the visible to vacuum ultraviolet spectral range. We present comprehensive numerical simulations to study the properties of coherent emission for a wide range of electron energies and bunch durations, including space-charge effects. These demonstrate that electron bunches with r.m.s. duration of 50 as, nominal charge of 0.1 pC and energy range of 100–250 MeV produce $10^9$ coherent photons per pulse in the 100–600 nm wavelength range. We show that this can be enhanced substantially by self-compressing negatively chirped 100 pC bunches in the undulator to produce $10^{14}$ coherent photons with pulse duration of 0.5–3 fs.

## Introduction

Several methods have been investigated for generating longitudinally coherent radiation from relativistic electron beams. These include coherent synchrotron radiation^1^, coherent transition radiation^2^, coherent Cherenkov radiation^3^, coherent undulator radiation^4^, free-electron lasers (FELs)^5,6^, FELs driven by pre-bunched electron beams^7^ and pre-bunched Cherenkov masers^8^. Such coherent sources are driven by ultra-short electron bunches with lengths or microstructure features shorter than the wavelength of the emitted radiation^9–13^. Conventional accelerators typically produce bunches with picosecond duration, or longer, and coherence is possible only in the microwave, terahertz or infrared spectral region^4,7,14,15^. However, if these long bunches contain small-scale internal structure, coherent emission can extend to shorter wavelengths, such as in the optical klystron^16^. FELs may produce bright coherent radiation from the far-infrared to the hard X-ray region by exploiting the ponderomotive microbunching that occurs during the interaction between electrons and radiation in a long undulator^17^. Microbunching can also be induced through the interaction between an electron beam and an intense laser beam in a modulator undulator^18^. Simulations indicate that trains of microbunches as short as 5 as (FWHM) can be produced, potentially paving the way towards attosecond or zeptosecond FELs^18–21^.

Laser-wakefield accelerators (LWFAs), on the other hand, have been shown theoretically^22,23^ and experimentally^24–27^ to directly produce electron bunches with durations of the order of a femtosecond. A LWFA driven FEL was first proposed in 2002^28^ and laser-driven synchrotron sources operating from the infrared^29^ to the VUV^30–32^ have also been demonstrated. Recent theoretical work indicates that bunches with duration of 100 as (FWHM), and possibly shorter, can be generated using a tailored plasma density profile^22,33,34^. Although the potential of LWFAs as drivers of coherent synchrotron sources has been considered before^28^, no systematic studies have been conducted so far. Here we present simulations of coherent emission in the visible to VUV spectral range by attosecond electron bunches traversing an undulator or wiggler. We investigate the characteristics of the emitted radiation and its dependence on electron beam parameters such as energy, energy spread and chirp. We also present start-to-end simulations of a laser-driven coherent synchrotron source.

## Results

### Undulator radiation

An electron traversing a linear undulator or wiggler emits radiation with wavelength

1 $$\begin{aligned} \lambda _1 = \frac{\lambda _u}{2\gamma ^2}\left( 1 + \frac{K^2}{2} +\gamma ^2 \theta ^2\right) , \end{aligned}$$

where $\gamma$ is the Lorentz factor, $\theta$ the observation angle, $\lambda _u$ the undulator or wiggler period and $K = e B_0 \lambda _u / (2\pi m_e c)$ the undulator parameter, where *e* is the electron charge, $m_e$ the electron mass, *c* the speed of light and $B_0$ the undulator peak magnetic field. Emission can also occur at odd harmonics of $\lambda _1$, on-axis, and both even and odd harmonics off-axis, within a cone angle $1/\gamma$. The power radiated by a bunch comprising $N_e$ electrons is

2 $$\begin{aligned} P = P_1\left[ N_e + N_e(N_e-1)f(\omega )\right] , \end{aligned}$$

where $P_1$ is the power radiated by a single electron and

$$\begin{aligned} f(\omega )=\left| \int {S(\vec {r})e^{-i(\omega / c) \vec {n}\cdot \vec {r}}}\,d\vec {r}\right| ^2 \end{aligned}$$

is the bunch form factor, with $\omega$ the angular frequency of emitted radiation, $S(\vec {r})$ the normalised bunch distribution, $\vec {n}$ the unit vector pointing from a beam particle to the observation point and $\vec {r}$ the vector pointing from the origin to the particle. When the bunch length is longer than the radiation wavelength, the form factor $f(\omega )$ is approximately zero and emission is mostly incoherent, with power proportional to the number of electrons $N_e$. On the other hand, when the bunch length is much shorter than the wavelength, $f(\omega )\approx 1$ on-axis, and emission is predominantly coherent, with power proportional to $N_e^2$. It is also possible for coherence to develop only off-axis ($\theta >0$), where the wavelength $\lambda _1$ of undulator radiation is longer^14^.

We investigated the generation of coherent undulator radiation from attosecond electron bunches using the software package GPT (version 3.4)^35–37^ and SPECTRA (version 10.2)^38–41^. A new module (GLmm) has been added to GPT to calculate coherent emission as the sum of longitudinal and transverse Laguerre–Gauss modes satisfying the paraxial Helmholtz equation^42^, as described in Methods. The mode amplitude is determined by the energy exchange between the particles and the radiation field, according to the energy conservation law. To explore the feasibility of an experimental demonstration, we use the undulator from the second section of the free-electron maser from the FOM-Institute for Plasma Physics in Rijnhuizen^43^. This has a period $\lambda _u={4}\,\hbox {cm}$, an undulator parameter $K=0.6$ ($B_0={0.16}\,\hbox {T}$) and $N_u=14$ periods, with antisymmetric end-poles. The on-axis wavelength $\lambda _1$ of undulator radiation is about 600 nm (2 eV photon energy) for 100 MeV electron energy and 100 nm (12.4 eV) for 250 MeV. Visible-VUV radiation can therefore be produced for electron energies accessible using laser-wakefield accelerators driven by 10–100 TW lasers^44^. Undulator radiation in the extreme ultraviolet and soft x-ray regions can be produced using 1–2 GeV electron beams, or an undulator with shorter period and at harmonics for larger *K* parameters. In general, short undulators and high electron energies are preferable to reduce beam degradation due to energy spread and space-charge forces, as discussed below.

### Coherent emission

**Figure 1:**
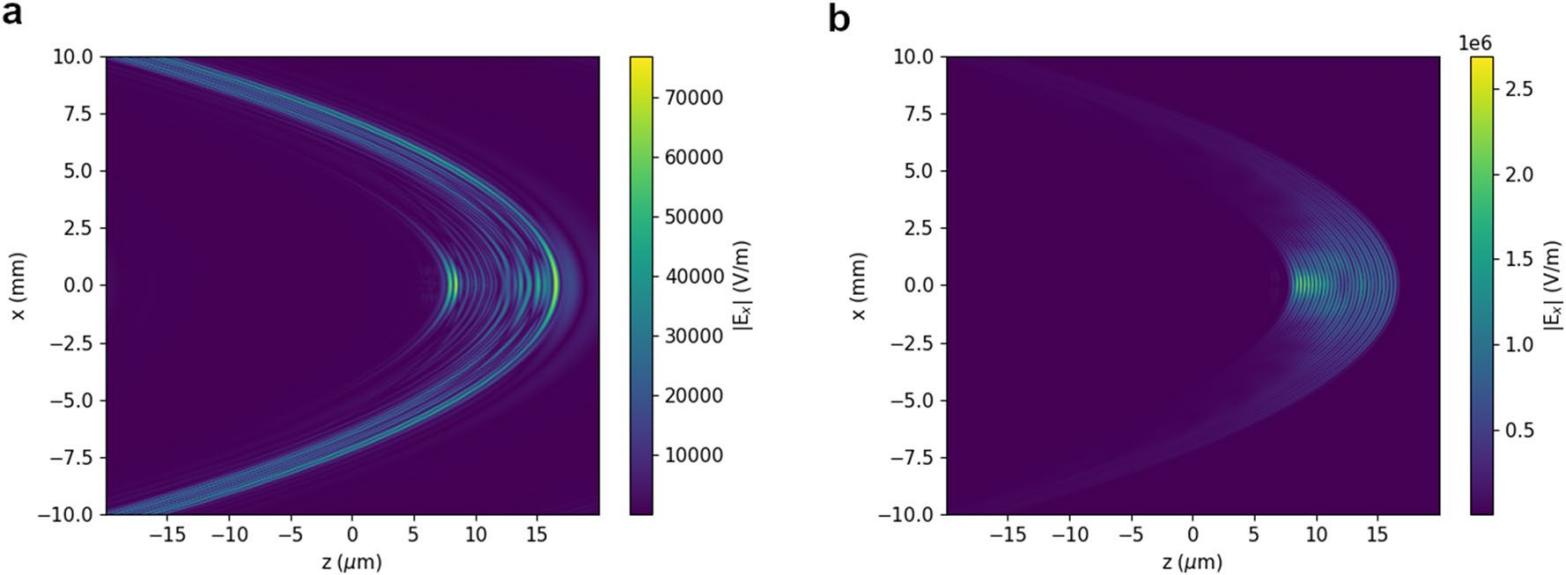
Electric field of undulator radiation calculated using GPT for an electron beam with 100 MeV energy, 0.1% uncorrelated energy spread, 0.1 mrad divergence, 1 $\pi$ mm mrad normalized emittance and 0.1 pC charge. The r.m.s. bunch duration is (**a**) 1 fs and (**b**) 100 as. The observation point is 1 m from the undulator exit.

The general characteristics of coherent undulator radiation are illustrated in Fig. 1, which shows a snapshot in the oscillation plane of the electric field generated by an electron beam with 100 MeV energy, 0.1% uncorrelated energy spread, 0.1 mrad divergence and 1$\pi$ mm mrad normalised emittance, corresponding to an on-axis undulator wavelength $\lambda _1\approx {600}\,\hbox {nm}$. Assuming a bunch with Gaussian shape both in longitudinal and transverse directions, the form factor is^14^

$$\begin{aligned} f(\omega ) = f_l(\omega ) f_t(\omega ) = e^{-\left( \frac{\omega }{c} \cos \theta \sigma _z\right) ^2} e^{-\left( \frac{\omega }{c} \sin \theta \sigma _r\right) ^2}, \end{aligned}$$

where $f_l$ and $f_t$ are the longitudinal and transverse form factors, and $\sigma _z$ and $\sigma _r$ are the r.m.s. bunch length and radius. On-axis ($\theta =0$), $f_l > 0.5$ when $\sigma _z < \lambda \sqrt{\log {2}}/ (2 \pi ) \approx 0.13 \lambda$. For a radiation wavelength $\lambda ={600}\,\hbox {nm}$, strong coherent emission is expected for bunch durations of 300 as or shorter. In Fig. 1a the r.m.s. bunch duration is 1 fs, which does not satisfy this condition, and coherence is mostly observed off-axis, where the wavelength is longer. Two spikes, however, are also visible on-axis, corresponding to coherent emission with a broad spectrum peaked at about 0.6 eV (2 $\upmu \hbox {m}$). This is due to the average velocity change when electrons enter and exit the undulator, in a process similar to edge radiation from bending magnets^45^. For a bunch duration of 100 as (Fig. 1b), on the other hand, coherence occurs over the full bandwidth and the field amplitude is strongest on-axis over the entire undulator length.

### SPECTRA simulations

**Figure 2:**
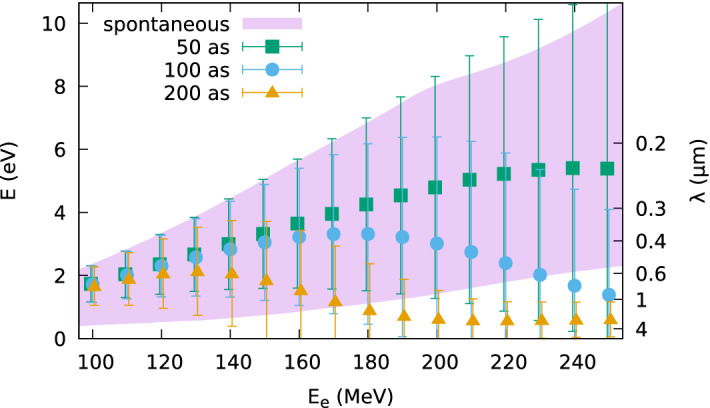
Mean photon energy *E* and r.m.s. bandwidth (error bars) of coherent undulator radiation produced by electron beams with energy $E_e$ between 100 and 250 MeV, 0.1% uncorrelated energy spread, 0.05 mrad divergence, 1$\pi$ mm mrad normalized emittance and r.m.s. bunch duration of 50 as, 100 as and 200 as. Radiation is observed 1 m from the undulator exit in a ${40}\,\hbox {mm} \times {40}\,\hbox {mm}$ area and integrated between 0.1 and 6 eV (${100}\,\hbox {MeV} \le E_e \le {150}\,\hbox {MeV}$), 10 eV (${160}\,\hbox {MeV} \le E_e \le {200}\,\hbox {MeV}$) or 15 eV (${210}\,\hbox {MeV} \le E_e \le {250}\,\hbox {MeV}$). The shaded area marks the r.m.s bandwidth $\sigma _E$ of spontaneous emission. Simulations carried out using SPECTRA.

**Figure 3:**
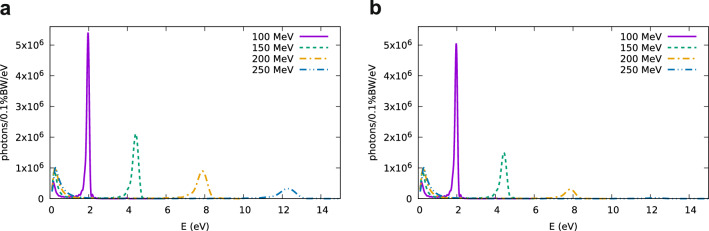
Sample spectra obtained for the parameters of Fig. 2, electron beam charge of 0.1 pC and r.m.s. bunch duration of (**a**) 50 as and (**b**) 100 as. Simulations carried out using SPECTRA.

Figure 2 shows the mean photon energy and r.m.s. bandwidth, represented by error bars, of coherent undulator radiation obtained with SPECTRA for electron beams with energy $E_e$ between 100 and 250 MeV, $0.1\%$ uncorrelated energy spread, 1$\pi$ mm mrad normalized transverse emittance and r.m.s. bunch duration between 50 and 200 as (120–470 as FWHM). The electron beam is quasi-collimated, with a waist located one period (4 cm) upstream from the undulator entrance, and divergence of 0.05 mrad, corresponding to an initial beam size of about 100 $\upmu \hbox {m}$ for 100 MeV and 40 $\upmu \hbox {m}$ for 250 MeV. This beam size and divergence is compatible with a laser-driven electron beam collimated using permanent quadrupole magnets^46^. A smaller beam size would be preferable to optimise the transverse form factor, but space-charge effects would be stronger. The bunch durations considered here require further advances in particle accelerator technology, as discussed later. Radiation is calculated 1 m from the undulator exit, integrated over a ${40}\,\hbox {mm}\,\times {40}\,\hbox {mm}$ area, which allows to capture low-energy off-axis radiation, and in a spectral range with minimum photon energy of 0.1 eV and maximum photon energy of 6 eV (${100}\,\hbox {MeV} \le E_e \le {150}\,\hbox {MeV}$), 10 eV (${160}\,\hbox {MeV} \le E_e \le {200}\,\hbox {MeV}$) or 15 eV (${210}\,\hbox {MeV} \le E_e \le {250}\,\hbox {MeV}$). The shaded area in Fig. 2 represents the r.m.s. bandwidth of spontaneous (longitudinally incoherent) undulator radiation integrated over the same area and between 0.1 and 15 eV. For a bunch duration of 50 as ($\sigma _z={15}\,\hbox {nm}$), the mean photon energy of coherent radiation initially increases with electron energy following the same trend as spontaneous emission, indicating coherence over the entire bandwidth. For electron energies higher than approximately 230 MeV ($\lambda _1\approx {115}\,\hbox {nm}$), however, the mean photon energy of coherent radiation flattens, indicating that the bunch is too long to achieve full coherence at the main frequency of spontaneous emission, whereas radiation at lower frequencies is still coherently enhanced. A similar drop is observed for 100 as ($\sigma _z={30}\,\hbox {nm}$) at electron energies above approximately 170 MeV ($\lambda _1\approx {210}\,\hbox {nm}$), and for 200 as ($\sigma _z={60}\,\hbox {nm}$) above 130 MeV ($\lambda _1\approx {360}\,\hbox {nm}$). This behaviour is also observed in the spectra shown in Fig. 3. The high-frequency peaks generated by 200 MeV and 250 MeV electron beams drop in magnitude when the bunch duration increases from 50 (Fig. 3a) to 100 as (Fig. 3b), whereas the low frequency part of the spectrum remains unchanged.

The r.m.s. divergence of coherent radiation generated by bunches with 50 as duration and 100 MeV electron energy is 0.66 mrad in the oscillation plane (*x*) and 0.72 mrad in the magnetic field plane (*y*). As expected, these divergences decrease when increasing the electron energy, to 0.25 mrad and 0.27 mrad, respectively, for 250 MeV. For bunches with 200 as duration, the divergence is approximately unchanged at 100 MeV, when the bunch is fully coherent, but grows to 3.6 mrad (*x*) and 5.1 mrad (*y*) for 250 MeV electron energy, where coherent enhancement is stronger off-axis, due to increase in the undulator wavelength with $\theta$ (Eq. 1). The pulse duration is determined by the radiation formation time as the electrons advance by one wavelength per undulator period^6,19^, resulting in a pulse with length $\lambda N_u$, corresponding to a duration between 5 and 30 fs for a 14 period undulator and radiation wavelength between 100 and 600 nm. However, shorter pulses are emitted if the bunch properties prevent coherent emission over the entire length of the undulator.

**Figure 4:**
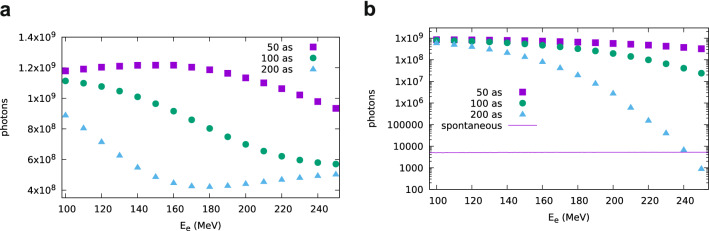
Flux of coherent undulator radiation obtained using SPECTRA for the parameters of Fig. 2 and electron beam charge of 0.1 pC. (**a**) Photons with energy $E>{0.1}\,\hbox {eV}$. (**b**) Photons with energy $0.9E_1< E < 1.1 E_1$, with $E_1$ the on-axis energy of spontaneous emission.

**Figure 5:**
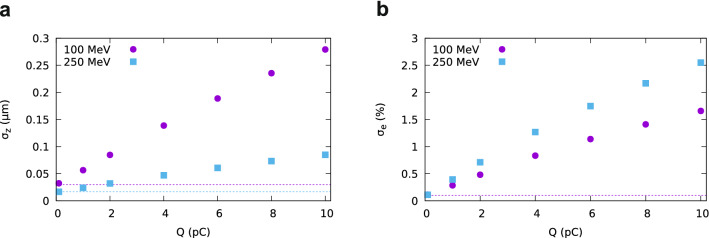
(**a**) Bunch lengthening and (**b**) energy spread growth induced by space-charge forces after 1 m drift in vacuum obtained using GPT for the parameters of Fig. 2 and an electron beam with r.m.s. bunch duration of 50 as and energy of 100 MeV and 250 MeV.

The integrated radiation flux per pulse (total number of photons emitted) has been calculated for the parameters of Fig. 2 and beam charge of 0.1 pC, which has been chosen to ensure that space-charge effects are negligible, as discussed below. The total number of photons produced by coherent emission, excluding the contribution of spontaneous emission, is presented in Fig. 4. For the electron energies, charge and bunch durations considered here, the number of photons per bunch is between $0.4\times 10^9$ and $1.2\times 10^9$ in the full bandwidth ($E>{0.1}\,\hbox {eV}$), as shown in Fig 4a. The number of photons in the high-frequency peak, however, quickly drops towards the level produced by spontaneous emission for 200 as bunch duration, as shown in Fig. 4b, which includes only photons with energy $0.9 E_1< E < 1.1 E_1$, where $E_1=h c / \lambda _1$ and *h* is Planck’s constant. A higher flux can be achieved by increasing the beam charge, but space-charge effects must be considered, which is not possible using SPECTRA. Simulations of the electron beam evolution performed with GPT for the parameters of Fig. 2 and initial bunch duration of 50 as indicate that space-charge effects are already significant for 1 pC, resulting in a bunch lengthening by about 1.9 times for 100 MeV and 1.5 times for 250 MeV after a 1 m drift in vacuum (Fig. 5a), while the energy spread increases to about 0.3% and 0.4%, respectively (Fig. 5b). This degradation in electron beam quality is more severe for higher beam charges and can suppress coherent emission. If the transverse size of the electron beam is increased, the strength of space-charge forces decreases, but coherent emission is weaker due to the transverse form factor^19^. Here we propose to mitigate space-charge effects using a chirped electron beam, lengthening the bunch and relying on ballistic self-compression inside the undulator to achieve the short durations required for coherent emission.

### GPT simulations

**Figure 6:**
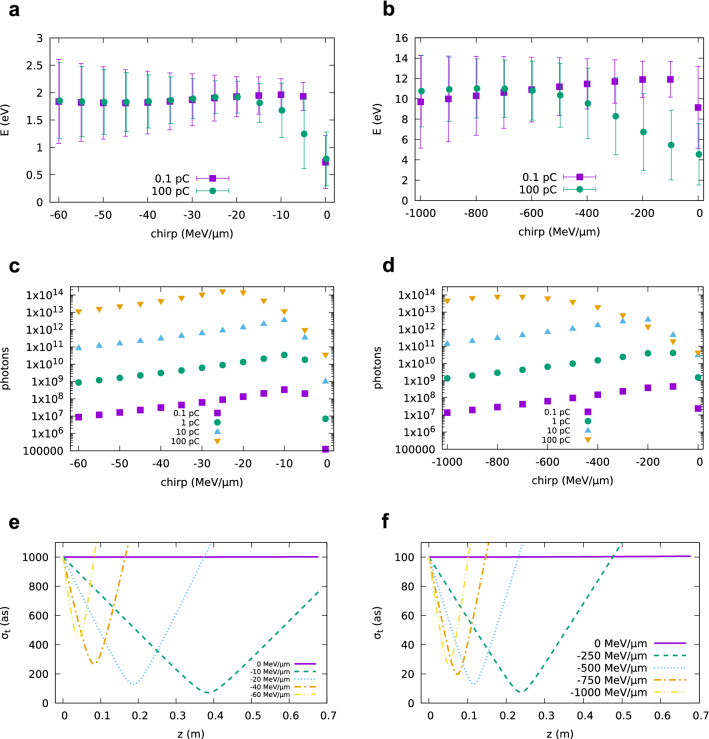
Undulator radiation obtained using GPT including space-charge effects for an electron beam with 0.05 mrad divergence, 1$\pi$ mm mrad normalized emittance, 0.1% slice energy spread, charge between 0.1 and 100 pC and varying linear chirp. Figures (**a**, **c**, **e**) show results for 100 MeV energy and 1 fs initial bunch duration. Figures (**b**, **d**, **f**) show results for 250 MeV energy and 100 as initial bunch duration. Radiation is calculated 1 m from the undulator exit in a 10 mm $\times$ 10 mm area. (**a**, **b**) Mean photon energy and r.m.s. bandwidth (error bars) of undulator radiation integrated between 0.1 and 10 eV. (**c**, **d**) Number of photons with energy $0.9 E_1< E < 1.1 E_1$. (**e**, **f**) GPT simulations showing the evolution of the bunch duration in vacuum for different chirp rates and space-charge off.

Coherent undulator radiation has been calculated using GPT including space-charge effects. Results for 0.1 pC charge have been found to be in excellent agreement with those from SPECTRA and the Lienard–Wiechert solver. Figure 6 shows the mean photon energy and the integrated number of photons in the range $0.9 E_1-1.1 E_1$ produced by an electron beam with charge between 0.1 and 100 pC, varying linear chirp, 0.05 mrad divergence, 1$\pi$ mm mrad normalized emittance. The energy is 100 MeV with 1 fs initial bunch duration (Fig. 6a, c), and 250 MeV with 100 as initial bunch duration (Fig. 6b, d). With no chirp, the bunch is too long for coherent emission at the main undulator energy $E_1$. With negative chirp, however, higher energy electrons initially towards the back of the bunch, ballistically catch up with the low energy electrons at the front, resulting in longitudinal bunch compression inside the undulator and coherent emission over the full bandwidth, as long as the bunch remains short. For charges up to 10 pC the highest flux is obtained when the bunch duration is shortest close to the centre of the undulator, as shown in Fig. 6e, f. For a charge of 100 pC, on the other hand, space-charge forces lengthen the bunch and reduce coherent emission as the bunch travels through the undulator. The highest flux is obtained for a chirp of about $-25$ MeV/$\upmu \hbox {m}$ for 100 MeV and $-800$ MeV/$\upmu \hbox {m}$ for 250 MeV, when the bunch duration is shortest at the entrance of the undulator, resulting in the emission of an intense burst of radiation containing about $10^{14}$ photons with duration (FWHM) of about 3 fs for 100 MeV and 500 as for 250 MeV. A comparison with simulations performed with space-charge turned off indicates that for this chirp rate space-charge effects slightly increase the bunch duration at the position of maximum compression, but subsequently slow down the rate of bunch lengthening, effectively keeping the bunch shorter for a few more periods and boosting the flux by about 20%. Furthermore, with no chirp a large energy spread can strongly reduce coherent emission, but these optimal chirp rates correspond to a total energy spread of about 10%. If the slice energy spread is increased from 0.1 to 1%, results are similar, but the flux decreases approximately by a factor of 5. GPT simulations performed without the undulator indicate that even for such high flux rate, the energy spread growth is dominated by space-charge forces, whereas the electron energy loss to radiation is small.

**Figure 7:**
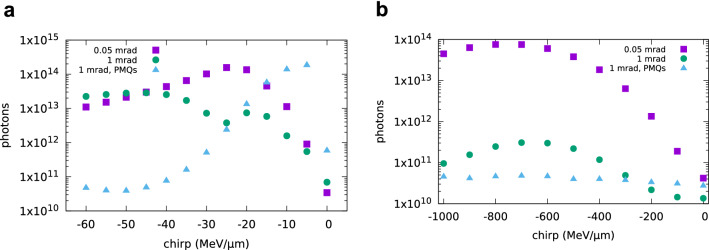
Undulator radiation obtained using GPT including space-charge effects for an electron beam with varying divergence, 1$\pi$ mm mrad normalized emittance, 0.1% slice energy spread, 100 pC charge and (**a**) 100 MeV energy and 1 fs initial r.m.s. bunch duration (**b**) 250 MeV energy and 100 as initial r.m.s. bunch duration. Radiation is calculated 1 m from the undulator exit in a 10 mm $\times$ 10 mm area.

It is not an insignificant challenge to produce chirped attosecond bunches with such characteristics, both from conventional and laser-driven accelerators. Unlike FELs operating in the saturation regime, a high degree of control is required to ensure that the intensity of the coherent source does not fluctuate significantly from shot to shot. LWFAs can produce electron beams with positive or negative energy chirp, depending on whether acceleration is stopped before or after dephasing^44^. The divergence, however, is typically at least 1 mrad, independent of the chirp rate, a value larger than in the previous simulations. Here we investigated two methods to match laser produced electron beams into the undulator. In one case, we used a triplet of permanent quadrupole magnets (PMQs)^47^ to collimate the electron beam, moving the undulator 20 cm from the accelerator. A second triplet could be used to focus the beam at the centre undulator. However, this would require moving the undulator further away and would introduce additional path differences, causing bunch lengthening. In the second case, we kept the undulator 4 cm from the accelerator and let the electron beam diverge inside. We performed GPT simulations to explore whether coherent emission in a space-charge dominated regime can be achieved under these more general conditions. Figure 7 shows coherent undulator radiation generated by an electron beam with 100 pC charge, 1$\pi$ mm mrad normalized emittance and varying chirp. Results obtained for a 100 MeV beam energy and initial bunch duration of 1 fs are presented in Fig. 7a. When the divergence is 1 mrad, and the beam is allowed to diverge inside the undulator, the maximum number of photons is about $10^{13}$ for a chirp rate of about $-50$ MeV/$\upmu \hbox {m}$. If PMQs are used to collimate the beam, the flux is enhanced to approximately $10^{14}$ photons, for a chirp rate of about $-5$ MeV/$\upmu \hbox {m}$, a value similar to the optimum chirp rate reported in Fig. 6c when space-charge effects are small. The pulse duration (FWHM) is about 3.5 fs in both cases. On the other hand, Fig. 7b shows that when the electron beam energy is 250 MeV, with initial bunch duration of 100 as, the flux is about 5e10 photons when using PMQs and 3e11 photons when the beam is allowed to diverge. No significant improvement is observed if the initial bunch duration is reduced to 50 as. We also performed GPT simulations for a beam with 1 mrad divergence and varying pointing, both in the horizontal and vertical plane. No significant differences are observed in the properties of coherent emission, but for pointing angles larger than 2 mrad the radiation beam is no longer fully contained in the chosen detection area. A more detailed study would have to be tailored to a particular experimental setup.

### LWFA-driven coherent synchrotron emission

Electron bunches with the properties required to produce coherent synchrotron radiation in the visible and VUV have not been demonstrated experimentally so far. Simulations indicate that conventional radiofrequency accelerators can produce trains of attosecond bunches using a modulator undulator^19^. Here we performed particle-in-cell (PIC) simulations with the code FBPIC^48^ to model a LWFA. The phase-space distribution of the resulting electron bunch was loaded into GPT to simulate coherent emission in the undulator. Typically, LWFAs produce femtosecond bunches, but attosecond durations can be reached using tailored plasma profiles where a small density bump triggers localised injection for a short time^22^.

**Figure 8:**
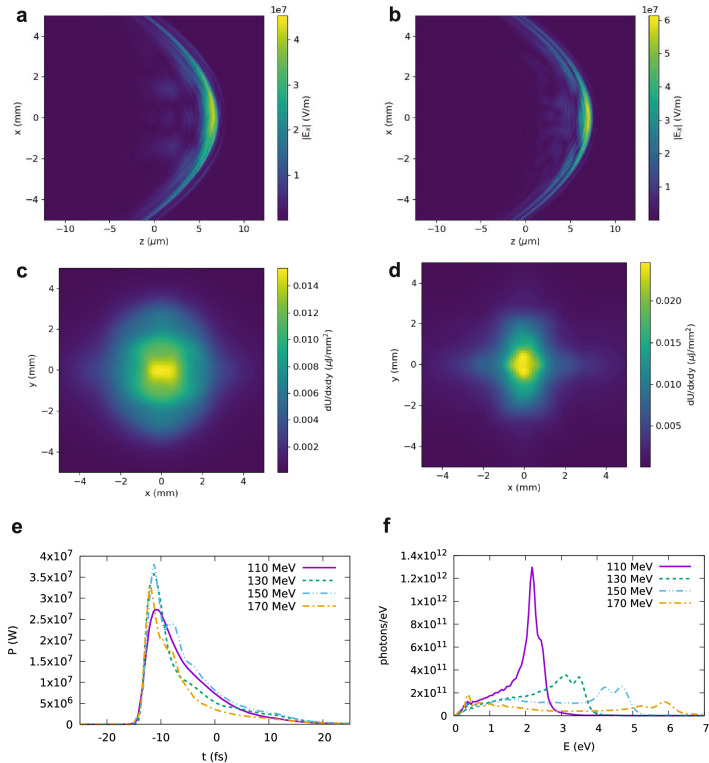
Undulator radiation obtained using GPT for a LWFA modelled using the particle-in-cell code FBPIC. (**a**) Electric field generated for 110 MeV mean energy and (**b**) 130 MeV mean energy. (**c**) Spatial distribution generated for 110 MeV mean energy and (**d**) 130 MeV mean energy. (**e**) Power and (**f**) spectrum for four electron beam energies. Radiation is calculated 1 m from the undulator exit in a 10 mm $\times$ 10 mm area.

PIC simulations are performed using a density profile characterised by a constant plateau with density of $2\times 10^{18}\,{\hbox {electrons}/\hbox {cm}{^3}}$ and a Gaussian bump located 1 mm from the plateau start, with amplitude of ${2.06\times 10^{18}}\,{\hbox {electrons}/\hbox {cm}{^3}}$ and width $\sigma _b={30}\,\upmu \hbox {m}$. The laser parameters are based on the 350 TW Ti:sapphire system at the SCAPA facility^49^ at the University of Strathclyde, which has a wavelength of 800 nm and a pulse duration of 25 fs. The chosen laser waist size is 20 $\upmu \hbox {m}$ and the normalised vector potential is $a_0=2.05$. Further details on the PIC simulations are provided in Methods.

After an acceleration length of 400 $\upmu \hbox {m}$ from the density bump, the resulting electron beam has a charge of 16.7 pC and a mean energy of 110 MeV with 5% energy spread. The slice energy spread is between 0.2 and 2.5% and the r.m.s. bunch duration is 320 as. After 700 $\upmu \hbox {m}$ acceleration length the mean energy has increased to 170 MeV with 4% energy spread and slice energy spread between 0.2% and 2%, with no change in bunch duration. The electron beam r.m.s. divergence is between 1.5 (170 MeV) and 2.5 mrad (110 MeV). Snapshots of the electron beam phase-space distribution taken between these positions have been loaded into GPT and sent through the undulator placed 4 cm from the accelerator exit to calculate coherent emission.

The radiation properties observed 1 m from the undulator exit are presented in Fig. 8. Snapshots of the electric field generated for an electron beam energy of 110 MeV and 130 MeV are shown in Fig. 8a and b, respectively. In both cases, coherent emission mostly occur in the first half of the undulator, before energy spread and divergence cause bunch lengthening and an increase in transverse beam size, which result in lower coherence. The corresponding spatial profiles are shown in Fig. 8c–d. The radiation r.m.s divergence is about 3 mrad for 110 MeV electron beam energy and 2 mrad for 170 MeV. Figure 8e shows the radiation power, which ideally should increase with the electron beam energy, as the radiation wavelength and pulse duration decrease. However, the bunch duration is not short enough to achieve good coherence at all selected energies, and no significant variation is observed. The radiation spectrum is shown in Fig. 8f. A clear peak is visible at the undulator energy $E_1$, with some broadening due to the energy spread. The amplitude of the peak decreases for increasing electron energy due to reduced coherence, as observed also in Fig. 3. The number of photons with energy $0.9E_1< E < 1.1E_1$ is between $9\times 10^{10}$ and $3\times 10^{11}$, and the number of photons in the full bandwidth is between $4\times 10^{11}$ and $9\times 10^{11}$. The relatively long bunch duration coupled to the larger energy spread and divergence result in a flux about two orders of magnitude lower than predicted by scaling the results of Fig. 4 for optimum conditions. It would be possible to decrease the bunch duration by reducing the size of the density bump and increasing the resolution of the simulations. However, the challenge is to develop plasma targets capable of producing such density profiles in the laboratory.

We have studied a prototype gas jet with design similar to^33^. PIC simulations have been conducted using a plasma density profile obtained from fluid dynamics simulations. The resulting electron bunch has an r.m.s. duration of 350 as, but the energy spread is large for the investigated parameters, because the density profile is not sufficiently flat. GPT simulations performed using the resulting electron phase-space distributions show that the bunch length increases quickly during propagation. Bunch sub-structure still leads to coherent emission in the visible and VUV spectral regions, but with lower photon flux and broader spectrum. Sub-structure is also observed in the electron beam distributions used to produce Fig. 8, but the good quality spectra obtained suggest that coherent emission should not be significantly affected when the bunch length is sufficiently short. However, further studies are required, ideally involving improved density profiles, but also experiments, since it is difficult to perform PIC simulations with sufficiently high resolution to finely resolve bunch sub-structure on attosecond time scales.

## Discussion

We have shown that electron bunches with r.m.s. duration of 50 as can produce coherent radiation in the visible–VUV spectral range with femtosecond pulse duration and about $10^{9}$ photons per pulse for 0.1 pC bunch charge. This corresponds to an energy of about 0.3 nJ at 600 nm and 0.8 nJ at 100 nm. If the charge is increased to 100 pC and the undulator is placed very close to the accelerator, the flux can be enhanced by up to 5 orders of magnitude using longer, negatively chirped bunches that ballistically self-compress during propagation. Perfect energy scaling is not achieved because chirp and space-charge effects limit coherent emission to a few periods close to the entrance of the undulator. Nevertheless, pulses with energy of ${30}\, \upmu \hbox {J}$ and duration of about 3 fs can be produced at 600 nm for several geometries. At 100 nm, pulses with energy of ${150}\,\upmu \hbox {J}$ and 0.5 fs duration are produced by a collimated beam, but the flux decreases by 3 orders of magnitude when using a diverging beam with or without permanent quadrupoles. Such flux levels are better than existing compact tunable sources in the UV^50^ and comparable to sources based on harmonic generation^51^. Using a much longer wavelength undulator the LWFA-driven coherent synchrotron source could be extended to the mid-infrared.

Start-to-end simulations of a LWFA-driven synchrotron source demonstrate the production of visible and UV radiation with flux of about $10^{11}$ photons. However, further experimental and theoretical work is required to fully assess the potential of LWFAs as drivers of high-quality coherent sources. Measurements of electron bunches with 1–10 fs duration have been reported, but attosecond bunches have not been produced in the laboratory so far. It is important to carry out experiments to validate and extend our theoretical predictions, because it is difficult to perform high-resolution PIC simulations over a wide parameter space. Experiments would also guide further advances in accelerator technology, which are necessary to generate bright coherent synchrotron radiation that outperforms existing visible and VUV sources. In particular, suitable plasma targets should be developed. If the plasma density profile can be controlled with sufficient precision, shorter bunch durations and smaller energy spreads than reported here should be possible. The use of higher plasma densities should be explored to boost the beam charge, which may also result in acceleration of a train of bunches to different energies. Plasma targets capable of chirp control would enable to maximize the flux, which is important for applications. Bunches with negative chirp are normally produced in a LWFA by accelerating the electron beam past the dephasing length, but this could result in the injection of additional bunches and in a growth of the slice energy spread, which would lead to bunch lengthening. Simulations presented here indicate that a moderate electron beam quality loss does not significantly impact on coherent emission in the visible and near UV spectral regions. For example, we have shown that intense coherent radiation can be produced using negatively chirped bunches with an initial duration of 1 fs. The generation of intense coherent radiation at shorter wavelengths, however, may require alternative schemes, such as multiple acceleration stages or plasma targets with improved density profiles. Bunch sub-structure should also be studied, since it can lead to the emission of ultra-short duration bright VUV radiation even for relatively long bunches. This could be advantageous, but have the side-effect of causing spectral broadening and large shot-to-shot fluctuations. The impact of electrons accelerated in the buckets further behind the laser pulse, or injected close to the exit of the plasma, should also be investigated. However, the energy should be lower and the bunch duration longer than for electrons accelerated in the first bucket, leading to coherent emission at lower photon energies, which can be separated using filters or a monochromator. A stable radiation source will also require advances in laser technology, since fluctuations in laser energy and spot size will translate into fluctuations in radiation wavelength and flux. When using chirped beams, the undulator can be made a few periods long, but for radiation sources driven by a LWFA the chirp rate may fluctuate from shot to shot and it may be preferable to use a longer undulator, which would ensure optimum compression at some point along the undulator, thus leading to more stable coherent emission.

With advances in accelerator technology to boost flux and stability, undulator radiation driven by attosecond electron bunches could be a very useful source of tunable coherent radiation over a broad spectral range. Possible applications include DNA damage studies^52^, photochemistry^53^ and astrochemistry^54^. Potentially, they could also be a building block for more advanced schemes, such as attosecond FELs. Furthermore, because coherent emission from a pre-bunched beam does not require the FEL instability to develop coherence, a much simpler, more compact and less demanding source can be developed: it only needs a short undulator and a relatively low brightness beam. Moreover, the coherent synchrotron undulator radiation from an appropriately shaped electron beam could be used as a seed to drive a compact FEL, reducing the requirement for very long undulators.

## Methods

### GPT simulations

The GLmm module has been added to GPT to calculate the interaction between particles and radiation using a decomposition in longitudinal and transverse Gauss–Laguerre modes, enabling simulation of coherent emission and amplification in FELs with no waveguides. This method is fast, supports arbitrary electron beam distributions, and enables study of the effect of space-charge forces. Simulations have been performed using 32,000 particles self-consistently interacting with 8 longitudinal and azimuthal modes. Results have been compared with those obtained using a custom Liénard–Wiechert (LW) solver, which supports radiation fields with arbitrary profiles, but is computationally demanding and does not include space-charge effects. In this case, electrons are propagated to the undulator entrance using GPT and the phase-space distribution is loaded into the LW solver. The trajectories in the undulator are calculated using an explicit embedded Runge-Kutta Prince-Dormand solver^55^ and the radiated spectral intensity is calculated by summing the contribution of each electron as described in^56^.

### SPECTRA simulations

Simulations have been performed using SPECTRA’s coherent emission module assuming a 6D Gaussian electron beam passing through a linear undulator with no end correction magnets. The beam waist location has been set 4 cm upstream from the undulator entrance. Beam parameters in SPECTRA are specified in the centre of the undulator, therefore the bunch lengthening induced by energy spread in the first half of the undulator has been calculated using GPT. Coherent emission has been calculated for a square slit detector aperture of ${40}\,\hbox {mm} \times {40}\,\hbox {mm}$ located 1 m from the exit of the undulator.

### FBPIC simulations

Particle-in-cell simulations of a laser-driven electron accelerator are performed using the quasi-3D code FBPIC. The box size is 60 $\upmu \hbox {m}$ in longitudinal direction (*z*) and 51 $\upmu \hbox {m}$ in radial direction (*r*), with a resolution of 17 nm and 100 nm, respectively. The number of azimuthal ($\theta$) modes is 3 and the number of macro-particles is $n_z=2$, $n_r=2$ and $n_\theta =12$, with cubic particle shape. The plasma is pre-ionised and the profile is given by a uniform distribution with density of ${2\times 10^{18}}\,\hbox {electrons/cm}{^3}$ with a Gaussian bump located 1 mm from the plateau start, with amplitude of ${2.06\times 10^{18}}\,\hbox {electrons}/\hbox {cm}{^3}$ and width $\sigma _b={30}\,\upmu \hbox {m}$. The laser beam is linearly polarised and has a wavelength of 800 nm and a temporal $\cos ^2$ shape with duration of 25 fs (FWHM of the intensity). The transverse profile is Gaussian, focused to a waist $w_0={20}\,\upmu \hbox {m}$ (in vacuum) at the entrance of the plasma. The laser normalised vector potential is $a_0=2.05$, corresponding to an energy of 1.5 J. The phase-space distributions of the resulting electron bunches contain about 32,000 particles. They have been loaded into GPT, including the particle weights, to calculate coherent emission.

## Data Availability

Data associated with research published in this paper is available at 10.15129/70f24ecf-d135-4c27-a650-31b040096803.
